# Performance of parallel FDTD method for shared- and distributed-memory architectures: Application tobioelectromagnetics

**DOI:** 10.1371/journal.pone.0238115

**Published:** 2020-09-11

**Authors:** Miguel Ruiz-Cabello N., Maksims Abaļenkovs, Luis M. Diaz Angulo, Clemente Cobos Sanchez, Franco Moglie, Salvador G. Garcia

**Affiliations:** 1 Department of Electromagnetics and Physics of Matter, University of Granada, Granada, Spain; 2 Hartree Centre S.16, Science and Technology Facilities Council, Daresbury Laboratory, Sci-Tech Daresbury, Keckwick, Daresbury, Warrington, United Kingdom; 3 Department of Ing. Automatica, University of Cadiz, Cadiz, Spain; 4 Dipartimento di Ingegneria dell’Informazione, Università Politecnica delle Marche, Ancona, Italy; King Abdulaziz University, SAUDI ARABIA

## Abstract

This work provides an in-depth computational performance study of the parallel finite-difference time-domain (FDTD) method. The parallelization is done at various levels including: shared- (OpenMP) and distributed- (MPI) memory paradigms and vectorization on three different architectures: Intel’s Knights Landing, Skylake and ARM’s Cavium ThunderX2. This study contributes to prove, in a systematic manner, the well-established claim within the Computational Electromagnetic community, that the main factor limiting FDTD performance, in realistic problems, is the memory bandwidth. Consequently a memory bandwidth threshold can be assessed depending on the problem size in order to attain optimal performance. Finally, the results of this study have been used to optimize the workload balancing of simulation of a bioelectromagnetic problem consisting in the exposure of a human model to a reverberation chamber-like environment.

## Introduction

Computational electromagnetics (CEM) has become an essential discipline which allows the analysis of large and complex engineering problems. Among the broad family of different numerical techniques found in CEM, the finite-difference time-domain (FDTD) method [1] is one of the most widely employed, being applied in many fields, including bioelectromagnetics, photonics, electromagnetic compatibility (EMC).

In essence, the FDTD method is an explicit marching-on-in-time algorithm based on the staggered space-time discretization of Maxwell’s curl equations [2]. In FDTD, the field unknowns are ordered spatially and are updated with their closest neighbors.

As a consequence of this explicitness, the algorithm can be easily parallelized to exploit the benefits of shared- as well as distributed-memory architectures [3]. Additionally, FDTD features good cache locality, which allows taking advantage of SIMD parallelization implemented in SSE and AVX instruction sets [4].

However, this explicitness also implies that making the next *snapshot* of the system requires the processing of all the electromagnetic field unknowns in the entire domain. Computationally, this means that all the unknowns must be moved between the RAM and the CPU at each time iteration. As all CPU cores on a computing node share a common addressable memory, this data movement creates a bottleneck in shared-memory access. This is the main performance-limiting factor, since it hinders making use of the CPU at full speed [5].

Therefore, while FDTD will scales almost linearly in multi-node distributed-memory clusters, its speeding-up saturates quickly inside each single-node shared-memory machine.

This work presents an exhaustive analysis of the effects of different memory bandwidths on the scalability and speed of a parallel FDTD algorithm with focus on: (i) memory-to-CPU for single-node performance of shared-memory parallelization with OpenMP and (ii) node-to-node distributed-memory parallelization with MPI. A noteworthy finding of this analysis shows that novel GPU-like processor architectures such as Intel’s Knights Landing help to alleviate memory bandwidth issues at zero cost of re-programming tasks opposed to native GPU architectures, which require considerable programming effort. Moreover, the results presented in this paper provide a quantitative estimate of the bandwidth threshold as a function of computational workload on different shared- as well as distributed-memory systems. Numerical experiments described in this work have been conducted on three different architectures: Intel’s Knights Landing and Skylake, and ARM’s Cavium ThunderX2.

Finally, we make a systematic study of its performance and apply it to a challenging problem consisting in the simulation, in a high performance computing (HPC) cluster, of the exposure of a human phantom to a statistically random EM environment.

## FDTD fundamentals

### Mathematical formulation

In brief, the FDTD method operates upon symmetric Maxwell’s equations [6]. In a simplified source-free form they are:

$$\begin{array}{c} -\nabla \times E=\sigma^{*}H+\mu \frac{\partial H}{\partial t}\;\nabla \times H=\sigma \;E+\varepsilon \frac{\partial E}{\partial t} \end{array}$$ (1)

where **E** and **H** are the electric and magnetic field vectors; all of these are functions of space and time (**r**, *t*). The parameters *ε*, *μ*, *σ* and σ* are the permittivity, permeability, and electric and magnetic conductivity of the medium.

The FDTD method introduced in [1–3, 7, 8] employs a second-order central-difference approximation for the space and time derivatives in Maxwell’s curl Eq (1) to yield an explicit marching-on-in-time procedure:

$$\begin{array}{c} \frac{\partial f(\nu ,\ldots )}{\partial \nu }\approx \frac{f\left(\nu +\frac{\Delta \nu }{2},\ldots \right)-f\left(\nu -\frac{\Delta \nu }{2},\ldots \right)}{\Delta \nu }. \end{array}$$ (2)

While FDTD was originally formulated for structured grids based on a stair-cased mesh resolving objects under study, it has also been expanded with geometry-conforming techniques [9, 10] to accurately model curved geometries.

For example, discrete finite-difference equations for electric and magnetic field components in 3D are:

Ex(i+12,j,k)n+1=CaEx(i+12,j,k)n+Cb(+Hz(i+12,j+12,k)n+12-Hz(i+12,j-12,k)n+12+Hy(i+12,j,k+12)n+12-Hy(i+12,j,k-12)n+12) (3)

Hz(i+12,j-12,k)n+12=DaHz(i+12,j-12,k)n-12+Db(+Ex(i+12,j,k)n-Ex(i+12,j-1,k)n+Ey(i,j-12,k)n-Ey(i+1,j-12,k)n), (4)

where subscripts *i*, *j*, *k* denote the spatial position and the superscript *n*—the time instant (*n*Δ*t*). The evolution constants *C*_*a*_, *C*_*b*_, *D*_*a*_, *D*_*b*_ are defined as [1]:

$$\begin{array}{cc} C_{a}=\frac{2\tau -\Delta t}{2\tau +\Delta t},\; & C_{b}=\frac{2\;\Delta t\;\tau }{\varepsilon \;\Delta \;(2\tau +\Delta t)}, \end{array}$$ (5)

$$\begin{array}{cc} D_{a}=\frac{2\tau^{*}-\Delta t}{2\tau^{*}+\Delta t},\; & D_{b}=\frac{2\;\Delta t\;\tau^{*}}{\mu \;\Delta \;(2\tau^{*}+\Delta t)}, \end{array}$$ (6)

where subscripts *i*, *j*, *k* denote the spatial position and the superscript *n*—the time instant (*n*Δ*t*). The evolution constants *C*_*a*_, *C*_*b*_, *D*_*a*_, *D*_*b*_ are defined as in [2]

$$\begin{array}{c} \tau =\frac{\varepsilon }{\sigma },\;\tau^{*}=\frac{\mu }{\sigma^{*}}, \end{array}$$ (7)

where Δ is the uniform spatial cell size. The evolution constants are location-dependent, but the spatial indices are omitted in Eqs (5), (6) and (7) for the sake of clarity.

**Fig 1:**
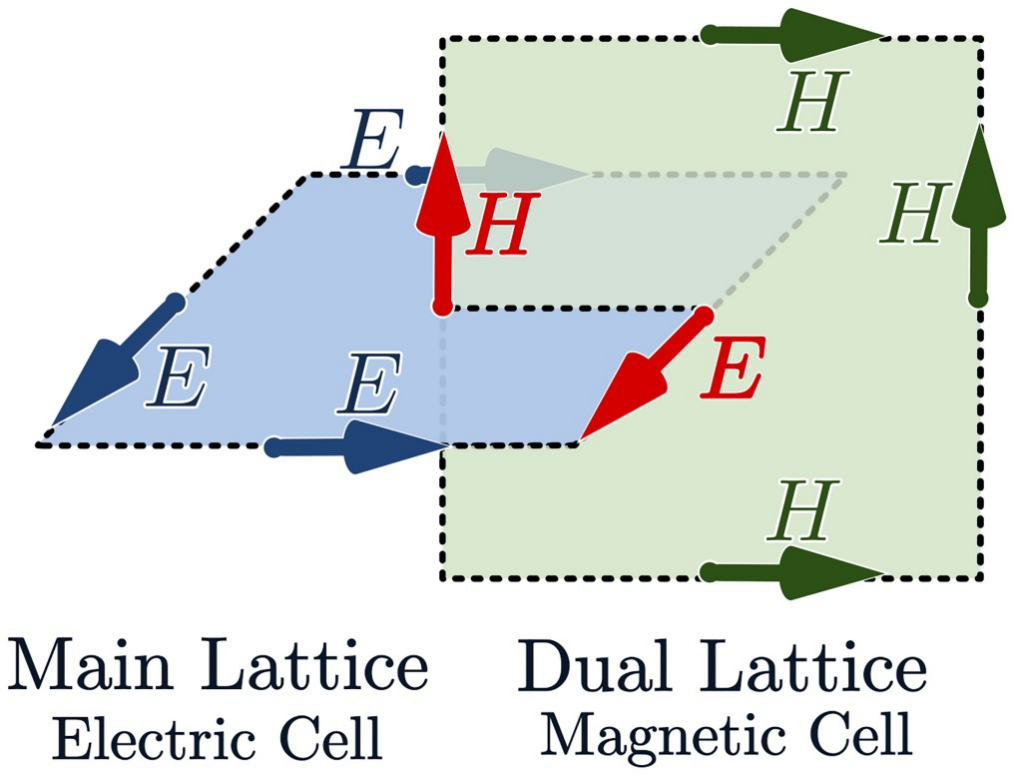
Arrangement of electromagnetic field components in a Yee’s cell. An FDTD mesh is formed by two lattices. Electric-field components are placed at the centres of cell edges of the primary lattice. Magnetic field components are located in a dual lattice, offset by half of a spatial step from the primary lattice.

The FDTD discretisation yields an explicit marching-on-in-time algorithm, where Cartesian components of electromagnetic fields **E** and **H** are naturally placed in the well-known staggered Yee’s grid arrangement (see Fig 1 for details) and evaluated at alternative time instants shifted by a Δ*t*/2 offset.

### Computer implementation

The FDTD stencil consists of the field values at the neighbouring points calculated at a preceding time instant (see Fig 1 for details). This feature makes FDTD a strong candidate to be efficiently parallelized at different levels: vector, shared- and distributed memory.

1. **Vectorization.** The standard arrangement of field components on a structured grid in FDTD gives an ordered placement of components in memory. Usually the field components are updated in the same order in which they are stored in memory. Therefore, a compiler can optimise the code using *vectorization* techniques based on a single-instruction-multiple-data (SIMD) paradigm [11]. The application of SIMD takes advantage of the aligned memory and the *cache memory spatial locality,* and therefore decreases the number of *cache misses.* Unfortunately, this is not straightforward for problems larger than the cache memory (typically in the order of dozens of MB). Performance suffers because all field components involved in a stencil update do not fit in the nearby memory addresses. These cache misses generate bottlenecks because of the breakdown of a continuous communication with the main memory. As a consequence, the overall simulation speed is limited by the *maximum memory bandwidth* between the cache and the RAM.
2. **Shared memory.** Modern CPUs feature large numbers of independent cores that share common address memory space. Explicit FDTD formulation implies that each field component within the spatial domain can be updated independently from the others. Computational space can be divided into subspaces, each of which is updated at the same time by different processing units. This kind of parallelism can be easily implemented applying *multi-threading* techniques based on *Fork-Join* procedures, such as OpenMP or C++11 threads.
3. **Distributed memory.** For problems larger than the shared-memory size, or in cases when speedup is limited by single-node memory bandwidth, distributed-memory clusters are of help. In this case the computational domain is divided into sub-domains and distributed across several independent *processes* usually assigned to different computers. Each process has its own memory space and the sub-domains are allocated in such a way that the neighbouring processes to share a common boundary. There is an overlapping region at the boundary, where the tangential magnetic fields are updated by both neighbours. At the end of each time step, only tangential *H*-field values need to be exchanged between the neighbouring nodes. These values suffice for calculation of *E*-fields to be performed at the next time step. Distributed-memory parallelism is commonly implemented with help of the MPI [12], which is designed for addressing, the memory distribution and the message passing, between processes, in distributed-memory architectures.

**Fig 2:**
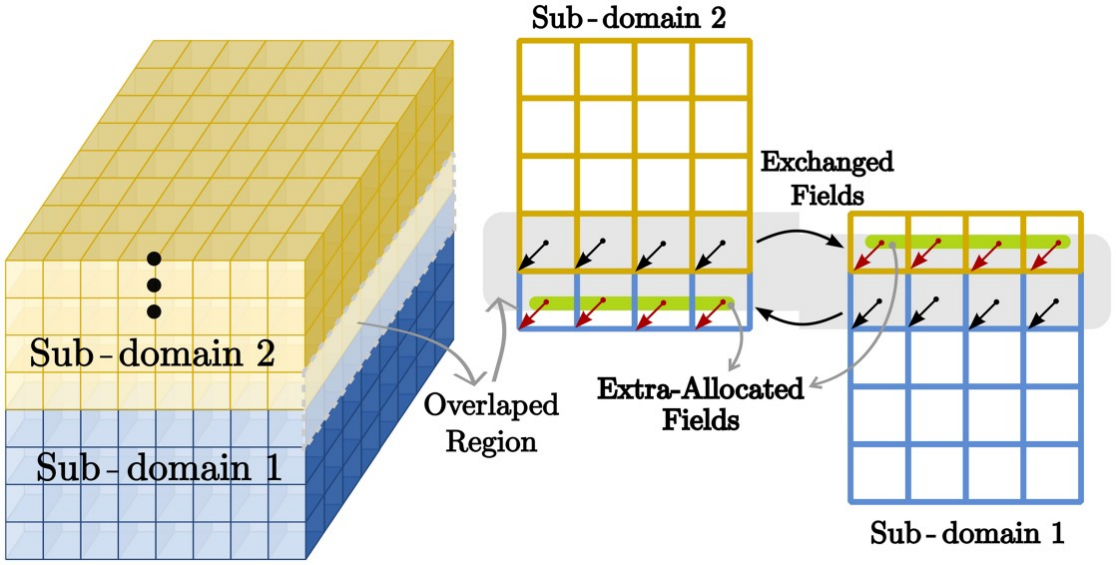
The computational domain is divided into sub-domains according to the number of available computer units. The H-fields in the overlapped region is exchanged between neighbour sub-domains.

SEMBA–UGRFDTD (http://www.sembahome.org) implements both shared- and distributed-memory parallelism based on the OpenMP and MPI tools [12]. The code has been refined and tested over the years on modern computers. Currently, SEMBA–UGRFDTD is applied by aeronautic companies for EMC assessment [13], and includes several enhancements to deal with complex problems [14]. Fig 2 illustrates the domain decomposition procedure implemented in this tool. Firstly, the computation domain is divided among different distributed nodes, that send and receive their portions of the data via MPI. Secondly, each compute node applies OpenMP to utilize its cores to advance the *E* and *H* fields.

**Fig 3:**
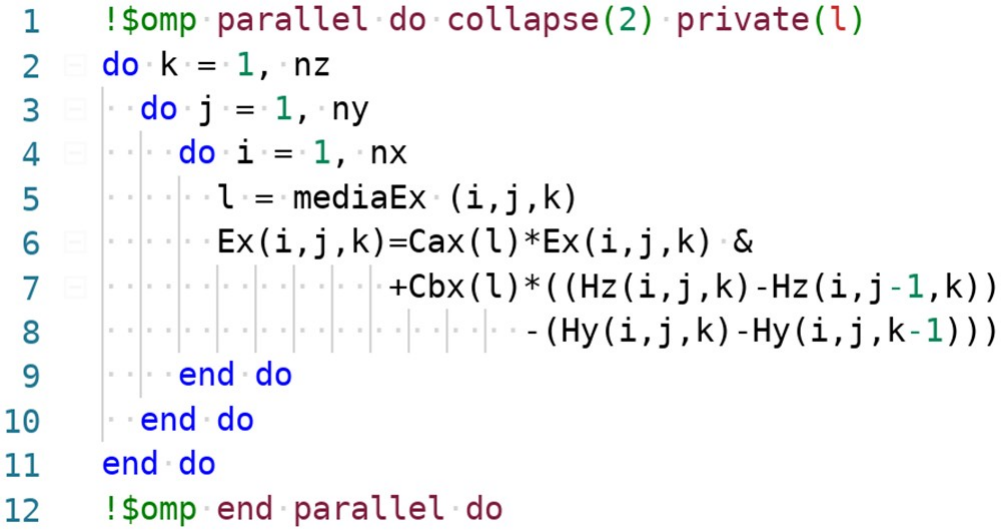
Fortran code to advance the *x*-component of the *E*-field. Piece of code extracted from the advancing routines of the SEMBA-UGRFDTD solver developed by the authors (http://www.sembahome.org).

Fig 3 presents an example code snippet for advancing the *E*_*x*_ field component over the entire spatial domain. the first line of the code contains an OpenMP sentinel to parallelize spatial loop traversal. The clause collapse(2) merges two top-level loops over *k* and *j* indices into one. Collapsing all three loops has been avoided, since this would hinder vectorization and thereby reduce the overall performance.

## Hardware platforms

### Knights landing

Intel’s Knights Landing (KNL) architecture has been enthusiastically welcomed by the stencil-based methods community. A unique feature of KNL that makes it stand out is the MultiChannel-DRAM (MCDRAM). MCDRAM with a maximum size of 16 GiB and a maximum bandwidth of 450 GiB/s may be setup in a *cache* mode [15]. The MCDRAM can operate as large cache with high throughput that favourably affects the FDTD performance.

The KNL-based supercomputer called Marconi was the first platform used for the FDTD performance experiments [16]. It forms part of the Italian supercomputing facilities CINECA. A KNL socket of Marconi consists of 36 tiles with 2 cores each, whereas each core features 2 vector-processing units. Marconi nodes are interconnected with the Omni-Path technology.

The UGRFDTD code was compiled with Intel Fortran compiler using the -O3 -xMIC-AVX512 compiler flags.

### Skylake

Another supercomputer used for the UGRFDTD performance tests was MareNostrum4 [17]. Each computing node of this system consists of 2 sockets featuring Intel Xeon Platinum (Skylake) 8160 CPU at 2.1 GHz with 24 cores each (resulting in 48 cores per node). The CPU L3 cache size is 32 MiB. Each node is equipped with 96 GiB of RAM (1.880 GiB/core). MareNostrum4 computing nodes are connected via the Intel Omni-Path network interconnect. SuSE Linux Enterprise Server is used as an operating system for MareNostrum4.

On MareNostrum4 the code was compiled with Intel Fortran compiler and Intel MPI mpiifort. The compilation flags used were -fopenmp -O3 -xCORE-AVX512 -mtune = skylake.

### ThunderX2

The last platform used for testing UGRFDTD code was an ARM-based Wombat cluster [18]. It consists of 16 compute nodes with 2 Cavium ThunderX2 CPU at 2.0 GHz per node. Each CPU has 28 cores resulting in 56 threads available for single-node computations. On ThunderX2 the caches are shared among groups of four cores. Each group has 4 MiB L3 cache slices, resulting in 1 MiB L3 cache per core. Each node has 256 GiB of RAM (4.571 GiB/core). EDR InfiniBand is used to interconnect the nodes. Wombat is operated by Red Hat Enterprise Linux 7.4 for 64-bit ARM architecture.

On Wombat the software has been compiled with the native ARM Fortran compiler and Open MPI. The compilation flags used were -fopenmp -O3 -armpl = parallel -mcpu = thunderx2t99.

## Numerical experiments

For an analysis of the performance of the FDTD method on shared- and distributed-memory architectures the UGRFDTD code was used. UGRFDTD is written in Fortran 95 featuring hybrid parallelization in OpenMP and MPI. For simplicity the UGRFDTD code utilized only generic update equations. No excitation sources, absorbing boundary conditions or any other extra features were used.

The problem-under-test consisted of a free space truncated by reflecting PEC (null E-field) conditions excited by initial conditions (given field values at *t* = 0). The simulation domain was discretised with a homogeneous isotropic Cartesian grid of cubic cells with the same number of cells along three Cartesian axes. For getting the memory bandwidth of the FDTD algorithm, we evaluate the amount of Bytes transferred between the main memory and the CPU, per unit of time in one FDTD iteration. For this aim, the used metric is the processing speed *V*_*p*_ defined as:

$$\begin{array}{c} V_{p}=\frac{{\text{S}}_{p}}{t} \end{array}$$ (8)

where *t* is the wall time in seconds required for the processing of one iteration and *S*_*p*_ is the total size to be processed in one iteration according to (3) and (4). The size to be processed at each time iteration is

$$\begin{array}{c} S_{p}=m\;p\;c\;v\;N_{\text{cells}} \end{array}$$ (9)

where *m* = 2^−30^ is a factor to attain speed in GiB/s, *v* = 6 is the number of variables in one update Eqs (3) and (4), *c* = 6 is the number of electromagnetic-field components per cell in the 3D FDTD method, *p* = 4B is the amount of memory required to store one variable in single precision, and *N*_cells_ = *N*_*x*_ *N*_*y*_ *N*_*z*_ is the number of cells.

Another quantity required for evaluating FDTD performance is the problem size *S*_*R*_ defined as:

$$\begin{array}{c} S_{R}=m\;p\;\left(c+q\right)\;N_{\text{cells}} \end{array}$$ (10)

where *q* = 6 is the number of components of the matrix with the index of the medium at each space location (one matrix per component).

The total simulation time of (8) can be predicted with a simple linear model [19]:

$$\begin{array}{c} t=t_{0}+t_{\text{CPU}}+\sum_{i}\{\begin{array}{cc} 0 & S_{R}<S_{i}\\ L_{i}+\frac{S_{p}}{{\text{BW}}_{i}} & S_{R}>S_{i} \end{array} \end{array}$$ (11)

where *t*_0_ is the fixed workload-processing time (this being independent of the problems size), *t*_CPU_ is the computing time taken by CPU, depending in turn on its CPU performance measured in FLOPS. Index *i* denotes the memory level (L1, L2, L3, MCDRAM or RAM), BW_*i*_ is the memory bandwidth and *S*_*i*_ is the size of each memory. The sum over *i* takes into account the effect of each cache-to-main-memory transfer time.

It should be noted that memory overhead is zero, if it is not occupied. Furthermore, within the limit of big sizes (*S*_*p*_ ≫ *S*_*i*_) substituting (8) into (11) *V*_*p*_ approaches the lower bandwidth *V*_*p*_ ∼ BW_lower_. On the contrary, for very small problem sizes *t*_CPU_ ≪ *t*_0_, and *S*_*p*_/BW_L1,L2,L3_ ≪ *t*_0_. Therefore *V*_*p*_ ∼ *S*_*p*_/*t*_0_, meaning that *V*_*p*_ grows linearly with *S*_*p*_.

The peak memory bandwidth (PMB) of the main memory can be calculated as,

$$\begin{array}{c} \text{PMB}=N_{\text{ch}}\;N_{\text{tr}}\;D \end{array}$$ (12)

where *N*_ch_ is the number of channels, *N*_tr_ is the number of transactions per second and *D* is the amount of data (in bytes) transferred per transaction (generally it is set in 8 B). For instance, the MCDRAM, is composed of 8 high-memory bandwidth units, meaning 8 channels with a speed of 7.2 GT/s with a block of 8 Bytes per transaction,

$$\begin{array}{c} \text{PMB}=8\;\times \;7.2\;\text{GiT/s}\;\times \;8\;\text{B}=460\;\text{GiB/s} \end{array}$$

One skylake socket is composed of 6 channels and has a speed of 2.667 GiT/s with block of 8 Bytes per transaction,

$$\begin{array}{c} \text{PMB}=6\;\times \;2.667\;\text{GiT/s}\;\times \;8\;\text{B}=128\;\text{GiB/s} \end{array}$$

therefore for two socket is 256 GiB/s.

### Single node

As an initial approach, a series of experiments evaluated the UGRFDTD performance on a shared-memory architecture. The software was run on single nodes of three different testing platforms: Marconi, MareNostrum, and Wombat. The code was launched using the maximum number of hardware cores per node, i.e. number of cores multiplied over the number of sockets: 64 for Marconi, 48 for MareNostrum and 56 for Wombat. See Table 1 for details. HyperThreading and analogous technologies were not utilized, since they did not improve the overall performance of the method.

**Table 1:** Comparison of platform specifications. TDP stands for Thermal Design Power and PMB for the Peak Memory bandwidth. Both values are given per socket.

	KNL	Skylake	ThunderX2
**Cores**	64	24	28
**Threads**	256	96	224
**Sockets**	1	2	2
**Base Freq**	1.4 GHz	2.1 GHz	2.0 GHz
**Turbo Freq**	1.6 GHz	3.7 GHz	2.5 GHz
**L3 Cache**	34 MiB	32 MiB	28 MiB
**TDP**	215 W	150 W	180 W
**PMB**	460 GiB/s	128 GiB/s	241 GiB/s

**Fig 4:**
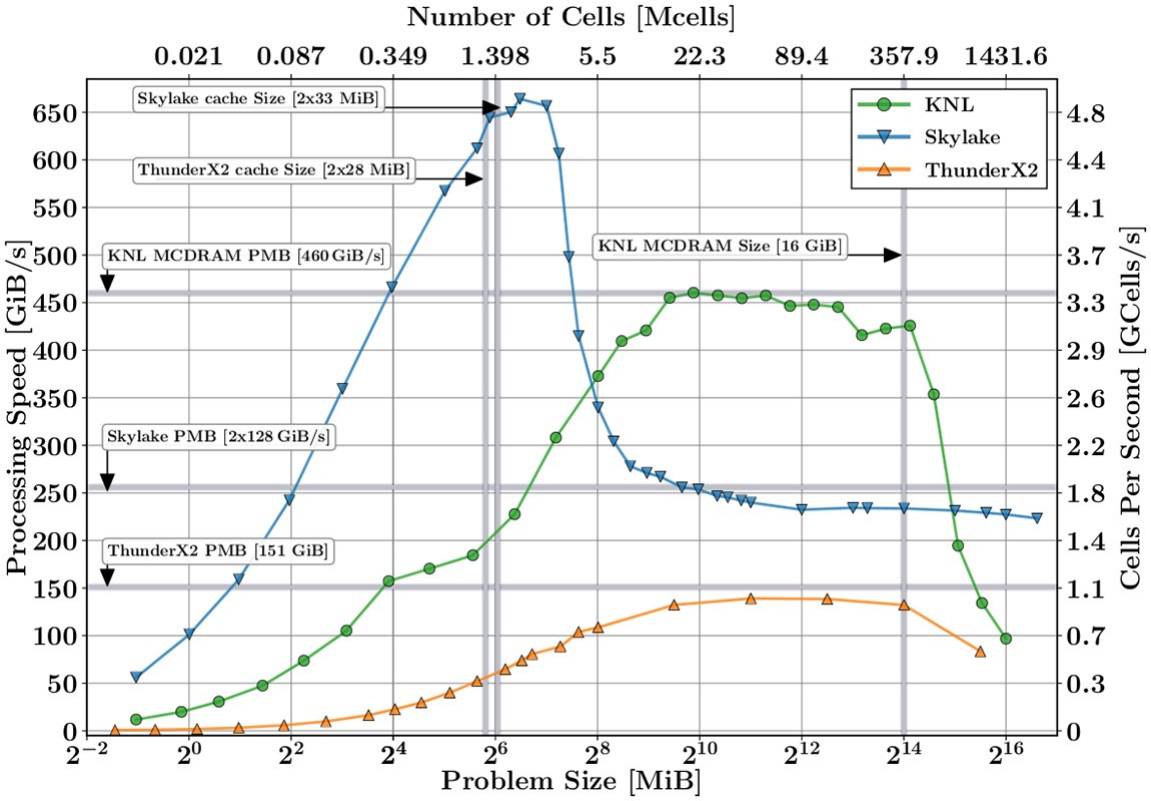
UGRFDTD performance on single nodes of Marconi, MareNostrum, and Wombat platforms. Memory sizes are delimited with vertical lines, and PMB with horizontal lines.

Fig 4 illustrates the processing speed *V*_*p*_ as a function of the problem size *S*_*R*_ for KNL, Skylake, and Cavium CPUs. According to this plot, three distinct performance regions can be outlined: (i) a problem size much lower than the cache size, (ii) a problem size comparable and fitting into the cache size, and (iii) a problem size much larger and overflowing the cache size.

In the first region (i), for small problem sizes, the processing speed has a strong influence on the latency *t*_*o*_ (OMP involves a significant overhead due to the fork-join procedures, which are independent of the problem size). However, as the problem size increases, there is more scope for the OMP parallelism, and the latency becomes less and less meaningful; hence, the processing speed is increasingly linear with the problem size, as predicted by (11) and (9). In this region, the memory hierarchy and the CPUs work optimally, since the memory bandwidth is higher than the CPU speed, and therefore the CPU speed is not limited.

The best performance for both KNL and Skylake architectures is achieved in the second region (ii), when the problem size is lower but comparable with cache size L3 for Skylake and MCDRAM for KNL. Since the BW_L3_ and BW_MCDRAM_ memories are much lower than their respective BW_*L*1, *L*2_, the more meaningful term in (11) is, respectively, *S*_*p*_/BW_L3_ and *S*_*p*_/PMB_MCDRAM_, in turn, the processing speed *V*_*p*_ (8) saturates in the memory bandwidth, respectively.

For KNL, the region (iii) starts when the 16 GiB MCDRAM is exahusted, and the RAM memory of the KNL begins to be occupied, with a subsequent drastic drop in its performance. As a result, the processing speed saturates at the bandwidth of its RAM (∼90 GiB/s). For Skylake, the region (iii) starts when its L3 cache size overflows. The performance plateaus at the PMB value of the its RAM memory (256 GiB/s).

In short, if we assume that a reasonable FDTD problem size is greater than 512 MiB, it becomes beneficial to utilize the KNL architecture when the problem size is smaller than the MCDRAM of 16 GiB. For problems larger than 16 GiB, Skylake CPUs outperform the KNL ones, and the processing speed *V*_*p*_ remains constant near the 250 GiB/s.

Fig 4 also shows the performance for ThunderX2 based on ARM64. It behaves qualitatively as Skylake: when the problem size is higher than the cache size, the processing speed saturates at the bandwidth of the its RAM memory (see Table 1). On the contrary, as expected, for small problems that fit within the cache size, the processing speed does not reach a maximum at the bandwidth of the cache. We deduce that the ARM compiler has not been used optimally, although further work for this would be needed, the main conclusions of this work remains unaffected.

In conclusion, multi-threading techniques produce for FDTD, a high degree of scalability in problem sizes smaller than the cache memory of the system. However, for problems larger than the cache size, multi-threading scalability is again limited by the maximum memory bandwidth of the system.

### Multiple nodes

**Fig 5:**
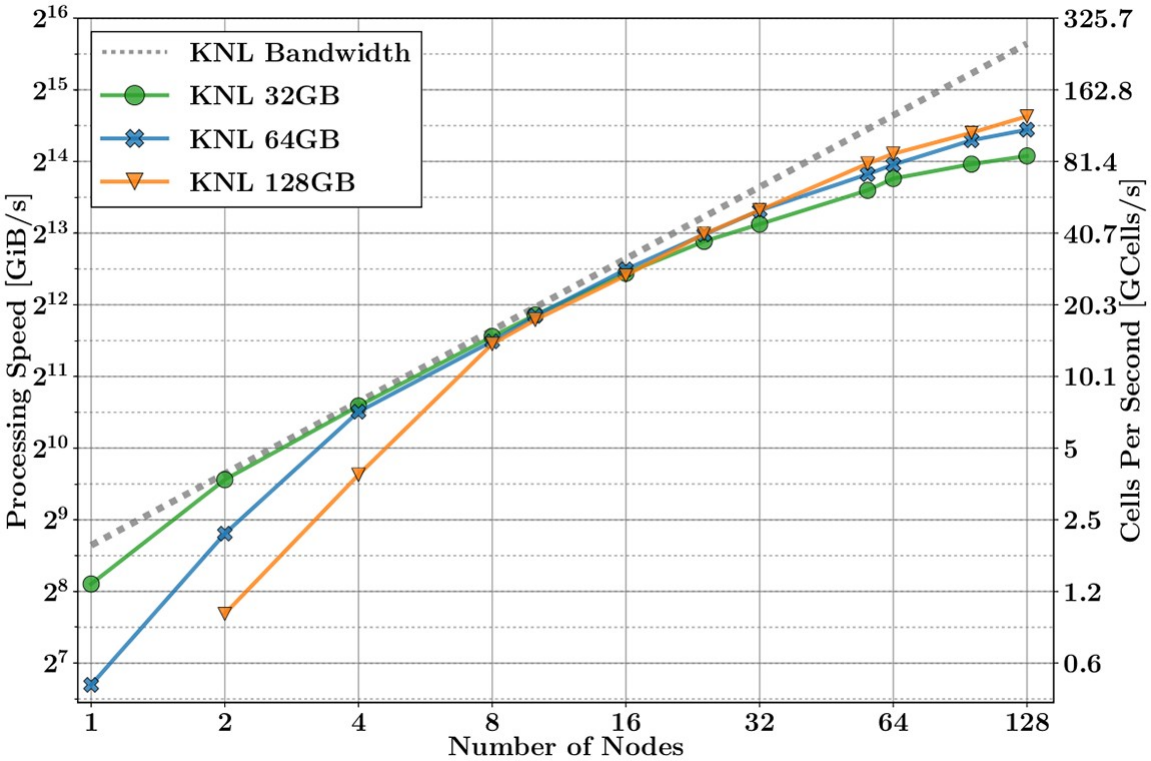
UGRFDTD performance on multiple nodes of Marconi platforms.

**Fig 6:**
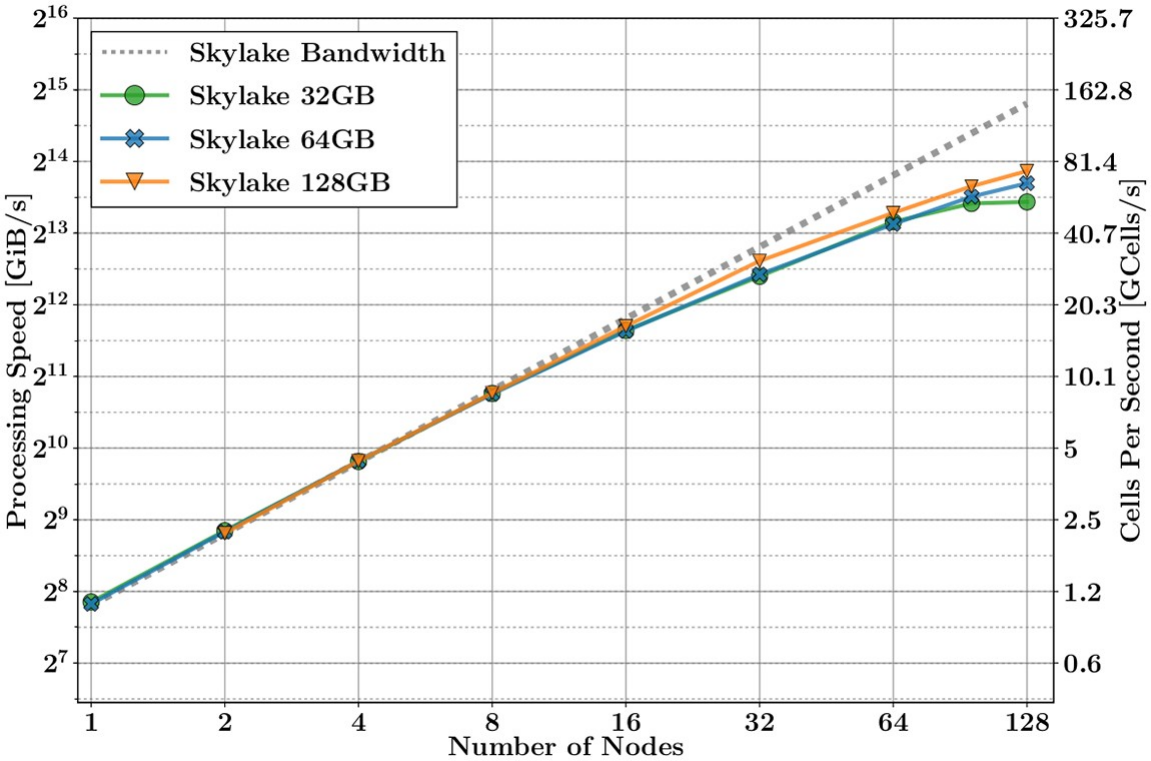
UGRFDTD performance on multiple nodes of MareNostrum platforms.

The limitation caused by the bottleneck of memory bandwidth can be overcome by increasing the number of independent compute nodes. To deal with this, we use HPC techniques based on a hybrid OMP-MPI methods. In this section, using this paradigm, we study the scalability of the processing speed as a function of the number of nodes, keeping the problem size constant. We have used the same test case as in the last section, for three different problem sizes: 32, 64, and 128 GiB. Figs 5 and 6, show the performance of the processing speed as a function of the number of nodes, for KNL and Skylake, respectively. The dotted grey line depicts the ideal scalability behaviour of the memory bandwidth for each platform.

According to the plots, two distinct performance regions can be outlined:

- Linear region scalability. This is the ideal region. The processing speed depends linearly on the PBW. The maximum processing speed is given by
  $$\begin{array}{c} V_{p}∼\frac{S_{p}}{L_{\text{mem}}+\frac{S_{p}/n_{\text{nodes}}}{{\text{BW}}_{\text{mem}}}}∼n_{\text{nodes}}\;\text{PMB} \end{array}$$
  where *L*_mem_ and BW_mem_ are, respectively, the latency and the bandwidth of the main memory. In the last approximation, we assume that the latency is negligible when *S*_*p*_ is big enough.
  Note from Fig 5 that KNL does not follow this linear trend at the beginning, because the problem size per node does not fit in the MCDRAM, and the processing speed is thus limited by the bandwidth of the RAM memory instead.
- When the scalability loses its linear trend. It happens when the MPI communication increases, and its latency becomes more meaningful. We can model it by adding the MPI overload in (11)
  $$\begin{array}{c} t∼L_{\text{MPI}}+\frac{S_{\text{com}}}{{\text{BW}}_{\text{MPI}}}+L_{\text{mem}}+\frac{S_{p}}{{\text{BW}}_{\text{mem}}} \end{array}$$ (13)
  where *S*_com_ is the problem size to be communicate via MPI (Fig 3) and *L*_mem_ is the MPI latency per communication.
  $$\begin{array}{c} V_{p}∼\frac{S_{p}}{L_{\text{MPI}}+\frac{S_{\text{com}}}{{\text{BW}}_{\text{MPI}}}+L+\frac{S_{p}}{\text{BW}}} \end{array}$$
  when *S*_com_/*BW*_MPI_ is very big respect to *S*_*p*_/BW_mem_ the processing speed saturates at the bandwidth of the MPI communications,
  $$\begin{array}{c} V_{p}∼BW_{\text{MPI}} \end{array}$$

### Application to bioelectromagnetics

In this section, we employ a complex bioelectromagnetics problem, to illustrate that the computer overload introduced by typical FDTD-simulation elements, including excitations, materials, on-the-fly post-processing…, with respect to the free-space case used above, does not change the main conclusions of the results drawn in previous sections, thus making access memory data the main figure-of-merit to evaluate the computer speed of a FDTD algorithm. The problem consist in a typical setup to simulate the exposure of a human phantom to EM fields. The CAD data has been provided by IT’IS from the virtual family [20] (codenamed as Ella v2.1), and it corresponds to a 1mm-resolution MRI scan of a 26 yo female, 1.63 m tall, with a weight of 72.4 kg. The phantom contains 22 different tissues whose conductivity *σ* and relative permittivity *ε*_*r*_ has been taken constant extrapolated from [21, 22] at 2 GHz (Table 2).

**Table 2:** Tissue relative permittivity and conductivity.

Tissue	*σ*(*S*/*m*)	*ε*_*r*_
Bladder	2.98	59.15
Bone	0.37	13.13
Brainstem	1.02	38.74
Cerebrum grey	1.51	49.69
Cerebrum white	1.02	38.74
Cartilage	1.42	39.76
Cerebellum	2.03	48.86
Gastrointestinal	2.98	59.15
Cerebrospinal fluid	3.04	68.54
Eye	3.04	68.54
Heart	2.03	60.16
Kidney	2.09	53.85
Liver	1.44	46.48
Mandible	0.37	13.13
Muscle	1.46	55.38
Skin	1.38	43.74
Skull	0.37	13.13
Respiratory system	1.03	36.50
Spinal cord	0.93	32.31
Thalamus	1.02	38.74
Tongue	1.46	55.38
Reproductive system	2.98	59.15

**Fig 7:**
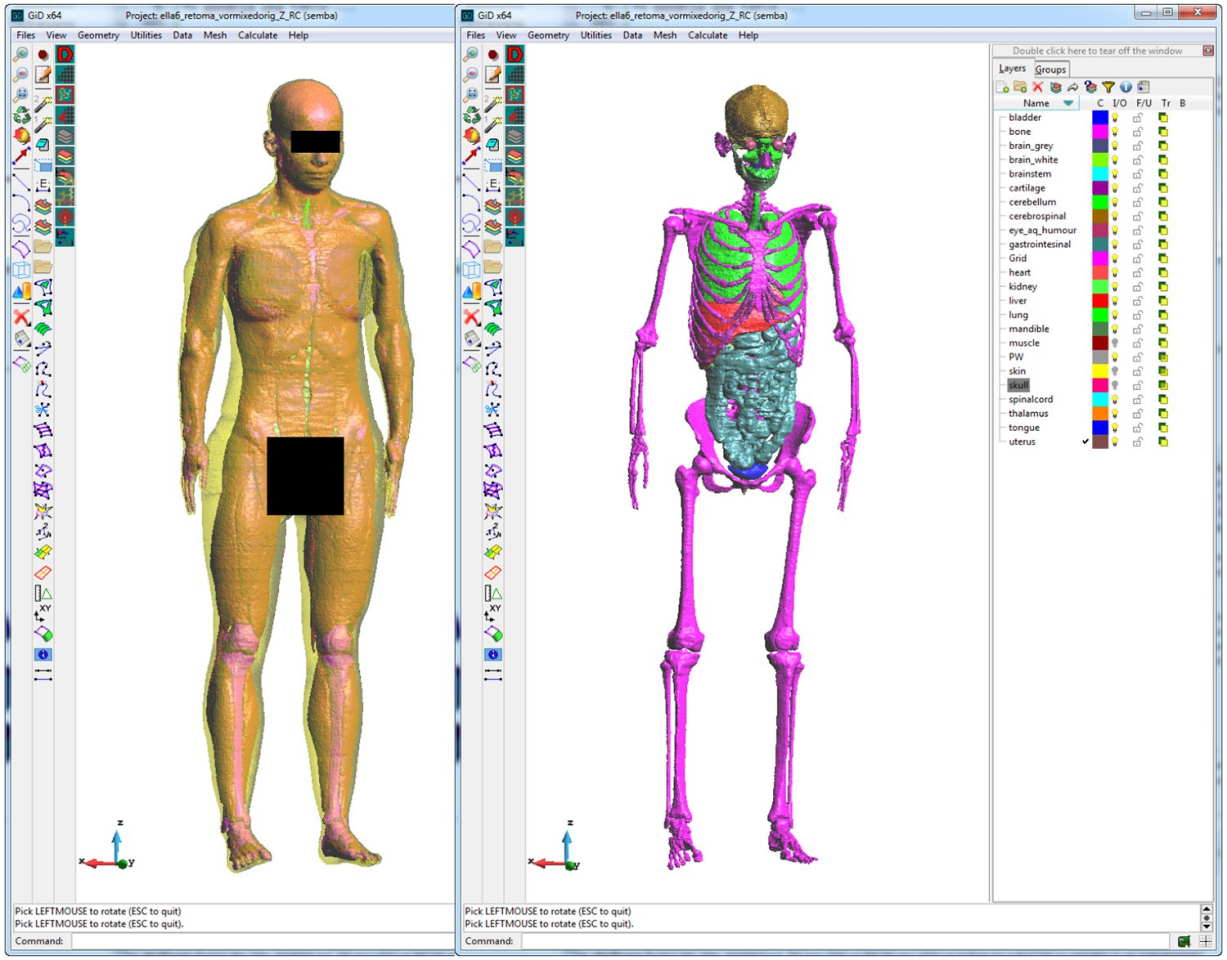
STL model of the Ella v2.1 prepared for simulation under SEMBA-UGRFDTD under the GiD tool (https://www.gidhome.com).

The model is provided as a set of files in stereolithography (STL) format (Fig 7 shows some details of the skin, muscles and internal organs pre-processed by the GiD tool (https://www.gidhome.com)). Prior to getting a FDTD mesh, we have encompassed a healing preprocessing stage to get manifold (water-tight) structures which have been remeshed into a mesh of triangles/tetrahedrons to finally yield a 1 mm cubic-voxel format, found by a Cartesian meshing tool embedded into the SEMBA-UGRFDTD solver, ending into a 540x320x1690 (0.3 GCells) model. This discretization requires for its simulation around 15 GiB, just below the 16 GiB fast-memory threshold of the Xeon Phi optimum zone of work, fitting into a single node. Speeds of around 3.3 Gcells/sec (450 GiB/sec) have been found. We have also conducted MPI scalability studies, but they do not present differences with classical Xeon MPI results which can be found in the literature [3]. We do not show them in this work, since we only wanted to focus ourselves on the performance of a realistic case in a single processor.

We have simulated the response of this human phantom under a statistical distribution of plane waves. This study is inspired in RC tests used in EMC, where it is a broadly-used standard [23], to assess the immunity of an electronic equipment.

The RC creates a good statistical EM distribution equivalent to illuminate the object with all directions and polarizations. It actually mimics its response in a real-life environment, where energy can come with such statistical uncertainty. The assessment of the biological effects of EM in RC has also been considered by a number of authors specifically for GSM frequencies, for the good field uniformity and the low impact that the insertion of the animal-under-test produces in the loading of the cavity to work with no degradation [24–26].

For our experiment, instead of meshing the RC with its whole actual complexity (stirrers, antennas, etc.), a simplified equivalent model from [27, 28] has been used. It employs a superposed set of plane waves, with a random uniform statistical distribution on their polarization, delays and direction of incidence, in a computational space truncated by perfectly matched layer (PML) absorbing boundary conditions [29], to simulate an ideally unbounded indefinite domain. Gaussian-modulated plane-waves were used covering the frequency range of interest. Since the focus of this paper is purely computational, and not to assess systematically the specific absorption rate [30] of the phantom, just data of the field levels and snapshots/animations of the EM fields inside the phantom are provided. A deeper study on the validity of this approach can be found in [31].

**Fig 8:**
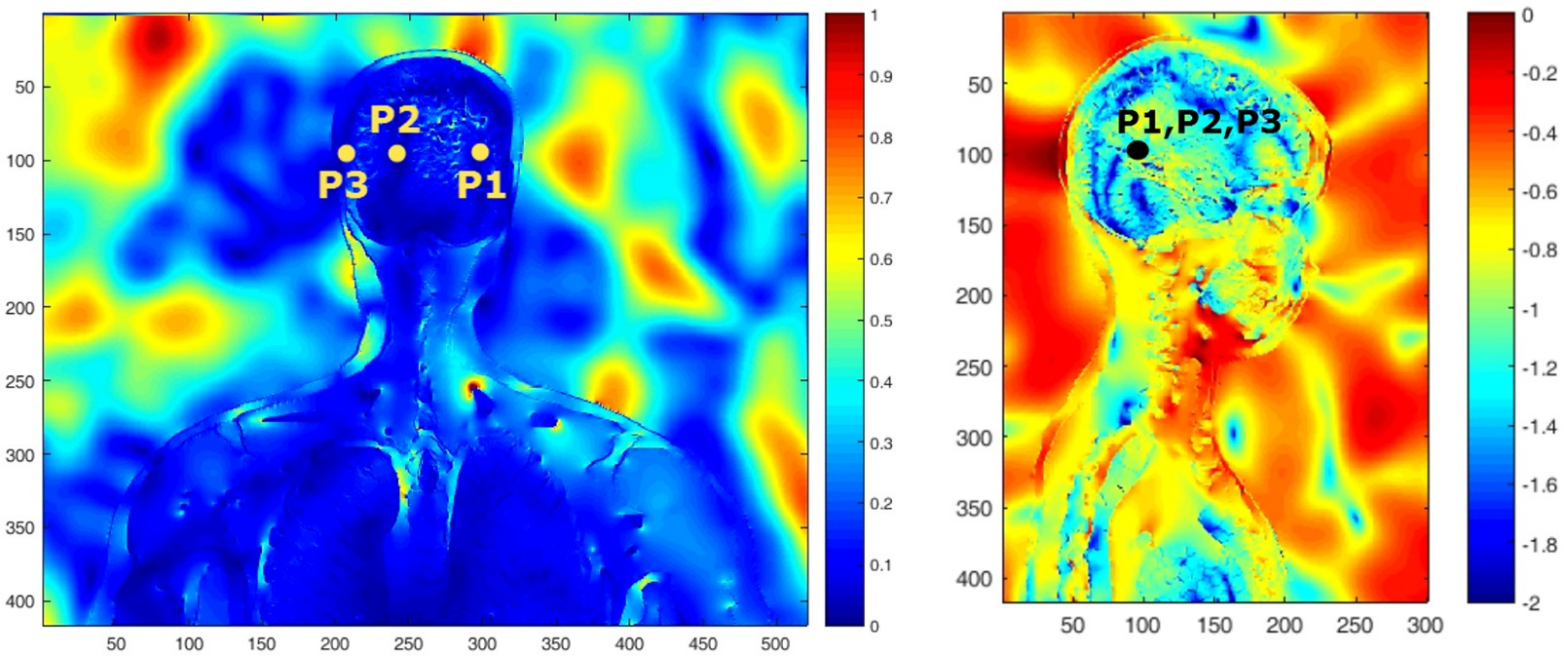
Two snapshots of the time-domain animations of the amplitude of the E-field normalized to the incident value: Frontal view with linear color scale (left), and side view with logarithm scale (right). The location of the 3 points for which the transfer function is calculated in Fig 9 are shown in black.

**Fig 9:**
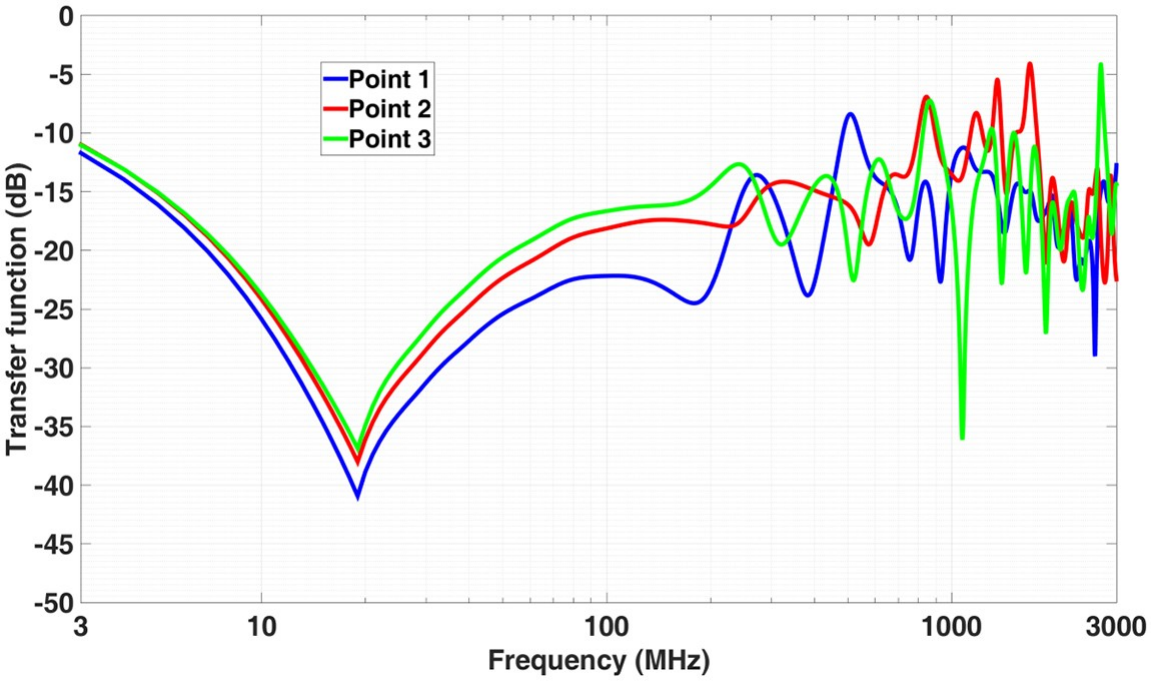
Transfer function at 3 different points inside the skull as a function of the frequency (see Fig 8 for their location).

The amplitude of the electric field has been evaluated at three observation positions inside the brain (see Fig 8). Fig 9 shows the modulus of the transfer function at each of them. For this, the E-field inside **E**_*t*_ is recorded in time, transformed into frequency and normalized by the incident E-field analytically calculated at the same observation point **E**_*i*_ (in the absence of the phantom).

$$\begin{array}{c} \left|T\right|=\frac{\sqrt{E_{t}\cdot E_{t}^{*}}}{\sqrt{E_{i}\cdot E_{i}^{*}}} \end{array}$$ (14)

where (*) denote the complex conjugate, and *i* identifies the probes.

Care must be taken to interpret the results in Fig 9. We have taken a constant conductivity and permittivity for the whole 3 MHz-3 GHz results, however this model is only reasonable between 1 GHz and 3 GHz where the variations of the constitutive parameters are not large. To predict with accuracy of the behavior in the whole band, high order dispersive models [32] should be employed. Note, for instance, that for the cerebrum white matter tissue, using the fitting provided by [33], we find at 3 MHz values differing almost one order of magnitude with respect to the constant ones used in this work *ε*_*r*_ = 285 and *σ* = 0.12 S/m.

## Conclusions

The goal of this study is to show how the performance of the FDTD algorithm is affected by the memory bandwidth. It is well known that the FDTD algorithm vectorizable can be readily parallelized and vectorized, due to its explicit formulation and memory locality. The performance of a CPU is usually measured in FLOPS by weighting its clock frequency, number of cores, vectorization instructions set, etc. However, we show that the maximum performance of the CPU cannot be reached when the problems do not fit into the cache memory. In this case, we demostrate that the performance is limited by the bandwidth between the socket and the different types of memories. Also, we show that the performance scales when number of distributed compute nodes increases, provided that the latency and bandwidth of the MPI communications do not dominate. The results can be extrapolated to any other method whose main computational burden resides in the memory access, rather than in the numerical calculus itself. A realistic simulation of a bioelectromagnetic case in a HPC cluster has served to validate the conclusions.
